# Bronchoalveolar lavage cytological alveolar damage in patients with severe pneumonia

**DOI:** 10.1186/cc3912

**Published:** 2005-11-25

**Authors:** Bogdan Grigoriu, Frédéric Jacobs, Fabienne Beuzen, Rony El Khoury, Olivier Axler, Francois G Brivet, Frédérique Capron

**Affiliations:** 1 Associate Physician, Department of Critical Care, Hôpital Antoine Beclere, Assistance Publique-Hôpitaux de Paris, 157 rue de la porte de Trivaux, 92140 Clamart, Paris, France; 2 Lecturer, Department of Critical Care and Pulmonary Diseases, UMF Iasi, Iasi, Strada Universitatii, 700000 Iasi, Romania; 3 Physician, Department of Critical Care, Hôpital Antoine Beclere, Assistance Publique-Hôpitaux de Paris, 157 rue de la porte de Trivaux, 92140 Clamart, Paris, France; 4 Physician, Department of Pathology, Hôpital Antoine Beclere, Assistance Publique-Hôpitaux de Paris, 157 rue de la porte de Trivaux, 92140 Clamart, Paris, France; 5 Physician, Department of Pathology, Hôpital Antoine Beclere, Assistance Publique-Hôpitaux de Paris, 157 rue de la porte de Trivaux, 92140 Clamart, Paris, France; 6 Physician, Department of Critical Care, Hôpital Antoine Beclere, Assistance Publique-Hôpitaux de Paris, 157 rue de la porte de Trivaux, 92140 Clamart, Paris, France; 7 Head, Department of Critical Care, Hôpital Antoine Beclere, Assistance Publique-Hôpitaux de Paris, 157 rue de la porte de Trivaux, 92140 Clamart, Paris, France; 8 Head, Department of Pathology, Hôpital Pitie-Salpetriere, Assistance Publique-Hôpitaux de Paris, 157 rue de la porte de Trivaux, 92140 Clamart, Paris, France

## Abstract

**Introduction:**

Histological examination of lung specimens from patients with pneumonia shows the presence of desquamated pneumocytes and erythrophages. We hypothesized that these modifications should also be present in bronchoalveolar lavage fluid (BAL) from patients with hospital-acquired pneumonia.

**Methods:**

We conducted a prospective study in mechanically ventilated patients with clinical suspicion of pneumonia. Patients were classified as having hospital-acquired pneumonia or not, in accordance with the quantitative microbiological cultures of respiratory tract specimens. A group of severe community-acquired pneumonias requiring mechanical ventilation during the same period was used for comparison. A specimen of BAL (20 ml) was taken for cytological analysis. A semiquantitative analysis of the dominant leukocyte population, the presence of erythrophages/siderophages and desquamated type II pneumocytes was performed.

**Results:**

In patients with confirmed hospital-acquired pneumonia, we found that 13 out of 39 patients (33.3%) had erythrophages/siderophages in BAL, 18 (46.2%) had desquamated pneumocytes and 8 (20.5%) fulfilled both criteria. Among the patients with community-acquired pneumonia, 7 out of 15 (46.7%) had erythrophages/siderophages and 6 (40%) had desquamated pneumocytes on BAL cytology. Only four (26.7%) fulfilled both criteria. No patient without hospital-acquired pneumonia had erythrophages/siderophages and only 3 out of 18 (16.7%) had desquamated pneumocytes on BAL cytology.

**Conclusion:**

Cytological analysis of BAL from patients with pneumonia (either community-acquired or hospital-acquired) shows elements of cytological alveolar damage as hemorrhage and desquamated type II pneumocytes much more frequently than in BAL from patients without pneumonia. These elements had a high specificity for an infectious cause of pulmonary infiltrates but low specificity. These lesions could serve as an adjunct to diagnosis in patients suspected of having ventilator-associated pneumonia.

## Introduction

Pneumonia is a common problem in critical care patients. Ventilator-associated pneumonia (VAP) complicates the course of as much as 27% of patients requiring mechanical ventilation [1,2]. Because mortality in patients with pneumonia is high and clinical studies have shown that an adequate antimicrobial treatment improves outcome, it is mandatory to identify infected patients precisely and give them effective treatment [3].

Unfortunately, at present there are no consensus criteria for the diagnosis of VAP [1,2]. Although clinical criteria (purulent tracheal secretions, fever), combined with the presence of new or worsening infiltrates on a chest radiogram or leukocytosis, are sensitive, they have poor specificity for diagnosing VAP [4]. Using solely clinical criteria entails a high risk of dispensing unnecessary antimicrobial treatment with the risk of emergence of multidrug-resistant organisms [5]. So far no biological test has proven useful in differentiating patients with and without pneumonia, despite very promising candidates [6]. Most diagnostic strategies rely today on quantitative cultures of samples of bronchial secretions from distal airways such as protected specimen brush, bronchoalveolar lavage fluid (BAL) or protected telescopic catheters (PTC) performed either under fibro-bronchoscopic guidance or in a blind manner. Even though there is still controversy over whether such techniques should be used as diagnostic criteria or just for guiding antibacterial treatment after diagnosis has been made, they are extensively recommended and used in clinical practice. Contrasting with the large amount of information about the quantitative culture of bronchial specimens, little has been published on the cytological aspects of BAL in pneumonia. Previous reports emphasized the lack of specificity of neutrophil predominance [7]. From all cytological information that can be gathered, so far only the presence of intracellular pathogens in more than 1 to 5% of BAL neutrophils has been retained as being highly suggestive of bacterial pneumonia in ventilated patients [8].

The lung response to local aggression after infectious pneumonia results in epithelial damage, edema, hemorrhage, the intra-alveolar accumulation of polymorphonuclear neutrophils and hyaline membrane formation. Macrophages that engulf red blood cells take the aspect of erythrophagocytes and later siderophages. The presence of one of these elements can be a useful marker of active alveolar hemorrhage [9]. Furthermore, damage at the epithelial alveolar level can lead to desquamation of dystrophic pneumocytes. These cells can eventually be found in BAL, as shown in some patients with acute lung injury [10]. Jacobs *et al*. [11,12] have described the presence of desquamated type II pneumocytes in the BAL from patients with various types of pulmonary infection. Thus, the presence of lesions of alveolar damage associated with alveolar hemorrhage and desquamated type II pneumocytes in BAL could help to differentiate pneumonia from other causes of pulmonary infiltrates (such as atelectasis or pulmonary edema) and could be of diagnostic importance.

The aim of our study was to evaluate the incidence of these findings in patients with severe pneumonia who were receiving mechanical ventilation.

## Materials and methods

A prospective study cohort was selected from patients hospitalized in our medical intensive care unit (ICU) between January 1999 and April 2002. Patients under mechanical ventilation for at least 3 days were selected in accordance with the following criteria: first, high clinical suspicion of pneumonia based on the presence of a new or worsening infiltrate on a supine chest X-ray, and at least two of the following: worsening pulmonary gas transfer as evaluated by the ratio of partial arterial oxygen tension to the fraction of inspired oxygen (PaO_2_/FiO_2_), a temperature of 38.5°C or more, purulent tracheal secretions, and leukocytosis; and second, no recent change (less than 3 days) in antimicrobial treatment. Patients with overt left ventricular failure were not included. For all these patients a fiber-optic bronchoscopy was performed and distal airways were sampled with a PTC (Combicath; Plastimed Lab, Le Plessis Bouchard, France) and a BAL was taken. Sampling conditions were described by Pham *et al*. [13] and were compliant with recent recommendations [14]. In brief, after the adjustment of FiO_2 _to 95% or more and deepened sedation if needed, the fiberscope was inserted (through an adapter piece permitting continuous ventilation) avoiding suctioning and without instilling local anesthetics.

The area of sampling was chosen on the basis of the chest radiograph and PTC was always performed before BAL was acquired. The volume of saline prepared for injection was 150 ml. On the basis of the results of the microbiological cultures, the patients were classified into two categories: those with a diagnosis of VAP in which at least one pathogen cultured at significant concentrations on PTC (10^3 ^colony-forming units/ml) or BAL (10^4 ^colony-forming units/ml) and those without pneumonia with no cultured pathogen in significant amounts, in whom no antimicrobial treatment was started. This second group provided the control cases with a high clinical suspicion of pneumonia but without bacteriological confirmation. Doubtful cases with pathogens cultured in non-significant concentrations were excluded from the final analysis. In parallel we recruited for comparative analysis a group of ventilated patients admitted to the ICU with a diagnosis of community-acquired pneumonia (CAP) in whom bronchoscopy with distal sampling was performed because of severity or rapid aggravation despite antibiotic therapy started in the previous 24 hours by their general physician or in the emergency room [15].

The ethical committee of our hospital approved the study protocol. This workup is standard in our unit and although informed consent was not mandatory, it was obtained from the patient's next-of-kin whenever possible.

### Data collection

The following variables were recorded from each patient: age, sex, Simplified Acute Physiology Score II (SAPS II) score [16] at admission and at the time of inclusion in the protocol, cardiac rate, arterial pressure, body temperature, Glasgow coma score before sedation, need for inotropic drugs, number of failing organs as evaluated by grade 3 and 4 SOFA (Sepsis-related Organ Failure Assessment) score [17], isolated microorganisms on quantitative microbiological cultures, time between admission and BAL, length of mechanical ventilation, length of stay in ICU, and status at hospital discharge.

### Specimen processing

After acquisition of BAL a small sample (20 ml) was sent for cytological analysis. PTC and the rest of the BAL were sent to the microbiology laboratory where they were immediately processed. For cytological analysis, slides were prepared by cytocentrifugation with a Shandon Cytospin^® ^4 Cytocentrifuge (Thermo Electron Corporation). Stains with May Grünwald Giemsa, Perls, Papanicolaou, Grocott and Ziehl were performed for each sample. The spot was examined at low and high magnifications; the dominant cellular population (more than 60%) and the presence of erythrophages and of desquamated alveolar type II pneumocytes were reported. Even if available, the result of the cytological examination was not immediately made known to the clinician and was not taken into account for patient management. The histologist was also blinded about the final diagnosis of the patient and the decision to treat or not.

### Statistical analysis

Results are reported as mean ± SD or as median and interquartile range when appropriate. Statistical comparisons were performed with Student's *t *test for variables with a known normal distribution. A Kruskal–Wallis test was used for multiple group comparison; group pairs were further analyzed with a Mann–Whitney *U *test. All *p *values were two-sided and *p *= 0.05 was considered significant. A Bonferroni correction was applied for multiple comparisons.

## Results

A total of 57 mechanically ventilated patients with clinical suspicion of hospital-acquired pneumonia were recruited during the study period. In 39 patients the diagnosis of VAP was confirmed and they received antibiotic treatment; in 18 patients the diagnosis of pneumonia was excluded and no antibiotics were started. In parallel we recruited 15 patients with severe CAP who fitted our inclusion criteria. The general characteristics of the population are described in Table 1. There were no significant differences between the three groups except for the delay between ICU admission and fibroscopy (*p *= 0.001, Kruskal–Wallis), which was statistically significantly shorter in patients without hospital-acquired pneumonia than in patients with VAP, as expected because the risk of pneumonia increases with the number of days under mechanical ventilation [18]. Our group of CAP had a very high mortality (80%) because they all required mechanical ventilation at admission, occurred in old patients (median age 73 years), seriously ill patients (mean SAPS II score at admission: 74.4) with a median of two failing organs (interquartile range 2 to 3). Consequently the length of stay in ICU was shorter because of a high early mortality rate in this group.

The isolated organisms from BAL and PTC show an usual profile for such infections, with a predominance of *Streptococcus pneumoniae *and *Haemophilus influenzae *in patients with CAP and *Pseudomonas aeruginosa*, *Staphylococcus aureus *and other Gram-negative rods in patients with hospital-acquired pneumonia (Table 2). The three *Candida *species isolated in patients with hospital-acquired pneumonia as well as the six isolated in patients without pneumonia were not considered to reflect active infection.

### Cytological analysis

**Figure 1:**
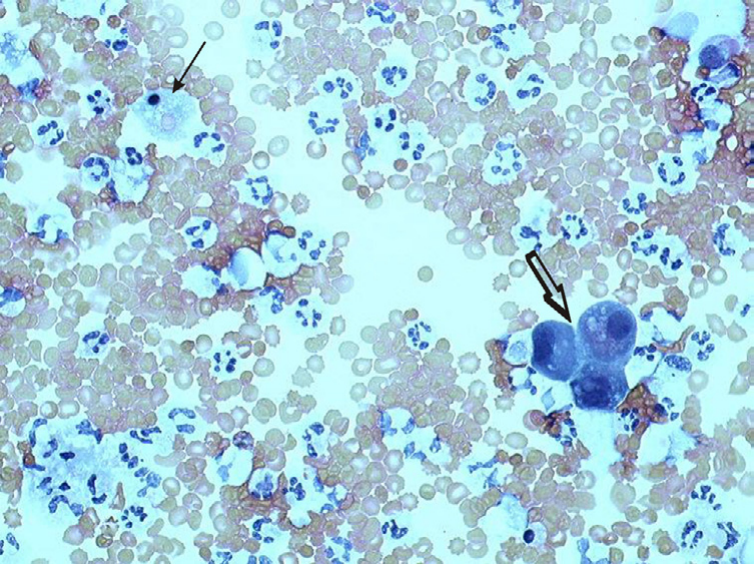
Bronchoalveolar lavage: predominance of neutrophil cell population, hemorrhage with erythrophagocytosis (black arrow) and desquamated type II pneumocytes (white arrow). Papanicolaou staining; magnification × 400.

The typical lesions observed are presented in Figure 1. In the pattern of cytological abnormalities we observed a predominance of neutrophil cell population in patients with pneumonia in most cases. However, as already reported [8], this element could not distinguish between patients with pneumonia and those without pneumonia because it was also present in about half of the latter group (Table 3).

Interestingly, the presence of erythrophagocytosis was noted only in patients with pneumonia, either hospital-acquired (13 out of 39 (33.3%)) or community-acquired (7 out of 15 (46.7%)). No such elements could be found in patients without pneumonia. Desquamated pneumocytes were observed much more frequently in patients with pneumonia (6 out of 15 (40%) in patients with CAP and 18 out of 39 (46.2%) in those with VAP) compared with ventilated patients without pneumonia (3 out of 18 (16.7%)). These two combined criteria were observed in 26.7% of patients with CAP and 20.5% of patients with hospital-acquired pneumonia (Table 3).

The estimated specificity and positive predictive values of the presence of erythrophagocytes alone or combined with the presence of desquamated pneumocytes are very high in our series (virtually 100% because there were no false positive cases). The sensitivity and negative predictive values are low (22.2% and 30%, respectively, for the whole group of all 54 patients with pneumonia). Calculated likelihood positive ratios were very high, stressing the possibility that such criteria could be used as an adjunct for a positive diagnosis (Table 4), thus helping to differentiate true infection from simple colonization.

## Discussion

Hospital-acquired pneumonia is a frequent and severe complication occurring in patients under mechanical ventilation. Rapid identification of such patients and accurate treatment selection are important goals for the clinician. Clinical criteria alone are not reliable enough to be used for diagnosis, resulting in unnecessary treatment. Moreover, bacterial colonization is frequently encountered, and it is often difficult to distinguish simple colonization from true infection. At present there are no consensual diagnostic criteria for VAP. Most strategies are actually based on a quantitative culture of samples from distal airways retrieved by BAL, protected specimen brush or PTC. The results of these cultures are available only after 48 hours. Thus quicker diagnostic criteria have been sought. So far only the presence of intracellular pathogens and more recently a high titer of soluble TREMs (triggering receptors expressed on myeloid cells) have been proven to be useful in diagnostic workup [6]. However, cytological analysis can provide other data of potential use as diagnostic criteria.

We show here that cytological lesions of alveolar damage expressed as the presence of hemorrhage and desquamated type II pneumocytes in BAL can be found in patients with severe pneumonia, either hospital-acquired or community-acquired. These findings occur much more frequently in patients with pneumonia than in those with other pathologies mimicking pneumonia. Our study was performed on a sample of patients admitted to the ICU with a high clinical suspicion of hospital-acquired pneumonia. This series consisted of very severely ill patients as shown by the high SAPS II score at admission, the number of failing organs and the high mortality. This sample may not be representative for all patients with VAP. However, because the pathological lesions of pneumonia should be similar whatever the severity of the disease, it is probable that similar lesions are to be found in all patients with pneumonia.

Another important point concerns the diagnostic approach used in our series. Our main goal in this descriptive study was to obtain a homogeneous sample of patients. We therefore selected very robust criteria for diagnosis, with the risk of excluding some patients with definitive pneumonia [14]. Because we excluded patients with recent changes in antibiotic therapy, these criteria are known to have an acceptable sensitivity and a good specificity for the diagnosis of VAP [19,20]. However, we cannot exclude the possibility that in some rare cases a misclassification that could result in reduced differences between groups.

A point worthy of discussion concerns the methodology of BAL and cytology examination. Actual recommendations suggest that volumes for BAL should be at least 120 ml [1] or 140 ml [14]. We did not monitor recovered volumes but used an instillation volume of 150 ml for our study, which is at the lower end of recommended values. If larger volumes were used it is possible that this could add to the sensitivity of the technique and reveal lesions of alveolar damage in more patients. Results are also highly dependent on accurate selection of the sampling area. It is possible that the large number of false negative cases in our series could be due to inaccurate selection of the sample area or low BAL volume.

However, the accuracy of cytological examination is dependent on the experience of the cytologist, which influences both the sensitivity and the specificity. This difficulty can be enhanced by the fact that there are no existing strict diagnostic criteria for such lesions. Nevertheless, all specimens were examined by the same team during the whole study. As a result the same diagnostic criteria were applied to all patients and reading was done blinded to the clinical characteristics of the patients, so it is improbable that significant reader-dependent bias occurred during the pathological examination.

Another potential bias could be introduced by the prolonged duration of the study. Long observational studies are subject to biases in recruitment criteria and to changes in diagnostic and therapeutic strategies. The monocentric nature of the study and the fact that it was necessary to include a sufficient number of patients imposed the duration of the study. However, using the same stringent criteria for selecting and classifying cases minimized potential biases.

An important point concerns the potential utility of the technique. Because these lesions are found combined only in about 20% of patients with pneumonia, their utility as a diagnostic criterion is confined to a subgroup of patients. Moreover, lesions of alveolar damage and therefore cytological alveolar damage have no specificity and can be found in various types of pulmonary injury, such as those induced by chemical agents or bacteria or even occur secondary to endothelial damage. However, the strong positive predictive value and the rapid availability of the results make them useful as an aid to the diagnostic decision and may help to differentiate infection from colonization. However, this implies that an 'on call' cytopathologist should be available at all times. It also should be noted that such lesions are found in some patients with acute lung injury irrespective of the presence of a pulmonary infection [10]. Our series included patients with severe pneumonia, bilateral infiltrates and very low PaO_2_/FiO_2_, and we show that these lesions were rarely found in patients without pneumonia. Therefore such criteria should be used with caution in patients with acute lung injury or acute respiratory distress syndrome, in whom more evaluation is needed.

## Conclusion

We showed that lesions of cytological alveolar damage are found in patients with severe hospital-acquired pneumonia and severe CAP much more frequently than in patients without pneumonia. Such lesions have a similar incidence in patients with severe CAP and VAP, confirming the robustness of our data. These findings may help in deciding whether isolated bacteria are the result of true infection. To the best of our knowledge similar data have not been published previously, except one report that showed a higher prevalence of reactive type II pneumocytes in patients with VAP [21]. However, the poor sensitivity and the necessity for a trained pathologist limits the potential utility of the technique. More prospective clinical evaluation is necessary before making recommendations for day-to-day practice.

## Key messages

• 	Cytological alveolar damage reflects active processes involving damage to alveolar epithelium.

• 	Although these lesions had no specificity in themselves, they can be found in both hospital-acquired pneumonia and CAP more frequently than arising from non-infectious causes of pulmonary infiltrates.

• 	These lesions could help to distinguish between colonization and true infection in patients suspected for VAP.

## Abbreviations

BAL = bronchoalveolar lavage fluid; CAP = community-acquired pneumonia; FiO_2 _= fraction of inspired oxygen; ICU = intensive care unit; PaO_2 _= partial arterial oxygen tension; PTC = protected telescopic catheters; SAPS = Simplified Acute Physiology Score; VAP = ventilator-associated pneumonia.

## Competing interests

The authors declare that they have no competing interests.

## Authors' contributions

FC, FGB and OA initiated the project; BG, FJ, OA, FB and REK retrieved the data and participated in patient management; and BG, FJ, FGB and FC analyzed the data. All authors were involved in drafting the initial manuscript and approved the final version.

## Figures and Tables

**Table 1:** Recruited patients

Group	Patients with community-acquired pneumonia	Patients with hospital-acquired pneumonia (*n *= 57)	
	With pneumonia	With pneumonia	Without pneumonia^c^
Patients, *n *(%)	15	39 (68.4%)	18 (31.6%)
Age, years (mean ± SD)	69.0 ± 17.4	65.3 ± 15.9	62.6 ± 19.7
Male gender *n *(%)	8 (53.3%)	26 (66.7%)	9 (50%)
Co-morbidities, *n*	8	25	10
Cardiac failure	0	8	2
Neoplastic disease	2	5	0
AIDS	2	2	1
Chronic respiratory failure	2	2	1
Cirrhosis	2	8	6
Ventilation, days before bronchoalveolar lavage^a ^(median (IQR))	1 (0–2)	8 (3–15)	3 (0–10.5)
Length of stay in ICU (median (IQR))	4.5 (1.0–15.7)	18 (10.7–34.0)	18 (7.5–30.2)
Mortality, *n *(%)	12 (80%)	21 (53.8%)	10 (55.6%)
SAPS II at admission (mean ± SD)	74.45 ± 21.50	64.36 ± 20.60	60.83 ± 21.41
SAPS II at inclusion (mean ± SD)	-	53.0 ± 19.4	66.0 ± 26.1
Vasoactive drugs (yes/no)	12/3	29/10	10/8
Number of failing organs^b ^(median (IQR))	2 (2–3)	3 (1–3)	2 (1–3)
None	0	7	2
1	2	6	5
2	8	6	3
3	4	11	5
4	1	7	1
5	0	1	2
6	0	1	0
Thoracic radiograph			
Bilateral opacities, *n *(%)	7 (46.7%)	20 (51.3%)	11 (61.1%)
Multilobar involvement, *n *(%)	11 (73.3%)	21 (53.8%)	13 (72.2%)
Clinical variables (mean ± SD)			
MAP, mmHg	68.11 ± 19.27	64.6 ± 16.6	61.55 ± 19.94
GCS before sedation	11.2 ± 5.0	9.85 ± 5.24	11.8 ± 4.9
GCS > 9	10	22	13
PaO_2_/FiO_2_, mmHg	177.91 ± 88.43	190.0 ± 63.3	195.27 ± 86.20
Creatinine, µmol/l	162.4 ± 66.4	198 ± 196	162 ± 122.2

^a^*p *= 0.027, Mann–Whitney *U *test between patients with and without hospital-acquired pneumonia. ^b^Sepsis-related Organ Failure Assessment (SOFA) score categories 3 and 4. ^c^The diagnoses for the 18 patients without ventilator-associated pneumonia were as follows: cardiac failure, 7 patients; sepsis with ARDS, 6 patients; cancer, 2 patients; atelectasis due to endobronchial lesion (cancer), 1 patient; non-significant growth of acinetobacter 102 (and not treated), 1 patient; no definite diagnosis, 1 patient. GCS, Glasgow coma score; ICU, intensive care unit; IQR, interquartile range; MAP, mean arterial pressure; FiO_2_, fraction of inspired oxygen; PaO_2_, partial arterial oxygen tension.

**Table 2:** Pathogens isolated by culture of bronchoalveolar lavage fluid or protected telescopic catheter

Species isolated	Patients with community-acquired pneumonia	Patients with hospital-acquired pneumonia (*n *= 57)	
	With pneumonia (*n *= 15)	With pneumonia (*n *= 39)	No pneumonia (*n *= 18)
*Streptococcus pneumoniae*	4	2	-
*Haemophilus influenzae*	3	1	-
*Legionella *sp.	1	-	-
*Klebsiella pneumoniae*	1	2	-
*Escherichia coli*	2	5	-
*Pseudomonas aeruginosa*	2	13	-
*Staphylococcus aureus*	1	9	-
*Proteus *sp.	-	3	-
*Enterobacter *sp.	-	2	-
*Serratia *sp.	-	1	-
Other	2	5	-
*Candida *sp.	-	3	6

**Table 3:** Cytological analysis of bronchoalveolar lavage fluid

Cell type	Patients with community-acquired pneumonia		Patients with hospital-acquired pneumonia (*n *= 57)		
	With pneumonia (*n *= 15)	*p*^a^	With pneumonia (*n *= 39)	No pneumonia (*n *= 18)	*p*^b^
Neutrophil predominance (%)	15 (100.0%)	0.274	36 (92.3%)	8 (44.4%)	0.001
Erythrophagocytes	7 (46.7%)	0.368	13 (33.3%)	0	0.006
Type II pneumocytes	6 (40.0%)	0.686	18 (46.2%)	3 (16.7%)	0.03
Erythrophagocytes and type II pneumocytes	4 (26.7%)	0.629	8 (20.5%)	0	0.04

^a^Mann–Whitney test for comparison between community-acquired and hospital-acquired pneumonias. ^b^Mann–Whitney test for comparison between patients with and without hospital-acquired pneumonia.

**Table 4:** Operational characteristics of studied criteria for the diagnosis of pneumonia

Criterion	All patients				Patients with hospital-acquired pneumonia			
	Se	Sp	PPV	NPV	Se	Sp	PPV	NPV
Presence of type II pneumocytes	0.444	0.833	0.889	0.333	0.462	0.833	0.857	0.417
Presence of erythrophagocytes	0.370	1.000	1.000	0.346	0.333	1.000	1.000	0.409
Both criteria	0.222	1.000	1.000	0.300	0.205	1.000	1.000	0.367

NPV, negative predictive value; PPV, positive predictive value; Se, sensitivity; Sp, specificity.
